# Plant Nutrient Contents Rather Than Physical Traits Are Coordinated Between Leaves and Roots in a Desert Shrubland

**DOI:** 10.3389/fpls.2021.734775

**Published:** 2021-10-26

**Authors:** Xiaoyan Jiang, Xin Jia, Shengjie Gao, Yan Jiang, Ningning Wei, Cong Han, Tianshan Zha, Peng Liu, Yun Tian, Shugao Qin

**Affiliations:** ^1^Yanchi Research Station, School of Soil and Water Conservation, Beijing Forestry University, Beijing, China; ^2^Key Laboratory for Soil and Water Conservation, State Forestry and Grassland Administration, Beijing Forestry University, Beijing, China

**Keywords:** functional trait, plant functional type, specific leaf area, specific root length, stoichiometry

## Abstract

Although leaf economics spectrum (LES) has been extensively tested with regional and global datasets, the correlation among functional traits of desert plants remains largely unclear. Moreover, examinations on whether and how leaf and root traits are coordinated have yielded mixed results. We investigated variations in leaf and fine-root traits across 48 species in a desert community of northern China to test the hypotheses that (1) the leaf-trait syndrome of plant species in desert shrublands follows the predictions of the global LES, and is paralleled by a similar root-trait syndrome, (2) functional traits related to nutrient contents and resource uptake are tightly coordinated between leaves and fine roots in desert ecosystems where plant growth is limited primarily by dry and nutrient-poor conditions, and (3) traits as well as their relationships vary among functional groups. Our results partially supported the LES theory. Specific leaf area (SLA) was correlated with leaf tissue density, phosphorus content, and carbon-to-nitrogen ratio, but not with leaf nitrogen content. Specific root length (SRL) was not correlated with other fine-root traits, and fine-root traits were largely independent of each other. Therefore, fine-root traits did not mirror the leaf-trait syndrome. Fine-root nitrogen and phosphorus contents, nitrogen-to-phosphorous ratio, and carbon-to-nitrogen ratio all increased with analogous leaf traits, whereas SRL was not correlated with SLA. After phylogenetic effects were considered, nutrient contents and their ratios still displayed stronger coordination between leaves and fine roots than did SRL and SLA. The overall pattern of trait variations and relationships suggested differentiation among functional groups. Our results suggest that despite the absence of a root-trait syndrome, fine-root functions in the studied desert community were probably coordinated with leaf functions with respect to nutrient allocation and use.

## Introduction

Leaf functional traits play an important role in plant carbon assimilation, water relations and energy balance (Ackerly et al., 2002), while root traits determine nutrient and water uptake that are crucial for plant survival and growth (McCormack et al., 2015; Weemstra et al., 2016; Wang et al., 2018). According to leaf and root economics spectrum (LES and RES, respectively), specific leaf area (SLA) and specific root length (SRL) are two key traits that indicate plant resource strategies (Wright et al., 2004; Cheng et al., 2016). A global foliar dataset indicated that 82% of total variance in photosynthetic capacity can be explained by SLA and leaf nitrogen content (LN; Wright et al., 2004; Ali et al., 2016). Specifically, species with high SLA exhibiting high LN, leaf phosphorus content (LP), high photosynthetic rate and short leaf lifespan, and low leaf tissue density (LTD), i.e., a resource-acquisitive strategy. The opposite is for species with low SLA exhibiting conserved traits (Wright et al., 2004; Pérez-Ramos et al., 2012). However, some studies found weak or a lack of correlation between SLA and other leaf traits across species (Zhou et al., 2010; Chen et al., 2013). In addition, existing studies on leaf traits mainly focused on forests and grasslands (He et al., 2008; Fajardo and Siefert, 2016; Hosseini et al., 2019), it remains largely unclear how leaf traits are correlated across species in desert communities.

Unlike leaves, the ongoing progress on fine-root trait correlations revealed a more complex and multidimensional economics space, reflecting a variety of evolutionary pressures and tradeoffs belowground (Kong et al., 2014; Xia et al., 2021). Some studies reported that fine roots of species with high SRL, small diameter, low tissue density (RTD), and high N content (RN) were associated with low construction costs, high respiration rates, and high turnover rates, a pattern analogous to leaf-trait correlations (Reich, 2014; Caplan et al., 2019). Exceptions to this pattern are nonetheless common (Holdaway et al., 2011; Weemstra et al., 2016). For example, Kramer-Walter et al. (2016) reported that SRL was independent of RTD and the plant economic spectrum across the most abundant tree species in New Zealand. Moreover, studies on temperate tree species showed no correlation between SRL and RN (Comas and Eissenstat, 2004) or between root lifespan and SRL or root diameter (Withington et al., 2006). Far less is known about whether there is a root-trait syndrome that parallels the leaf-trait syndrome in desert communities.

For a plant economics spectrum to occur, traits of different plant organs (e.g., leaf and root) must be coordinated in a way that follows evolutionary and biophysical constraints (Reich, 2014; Carvajal et al., 2019). The level of coordination between root and leaf traits can be multidimensional, meaning that certain root traits are coordinated with analogous leaf traits, while other root traits vary independently of leaf traits (Kramer-Walter et al., 2016). Empirical evidence indicates that analogous leaf and root traits can be correlated across species in grasslands on the Inner Mongolian Plateau and the Tibetan Plateau (Geng et al., 2014). However, other studies showed that analogous leaf and root traits were weakly correlated at the global scale (Craine et al., 2005) and that the same plant can have aboveground traits that are correlated with root traits of the opposite growth strategy in temperate grasslands (Personeni and Loiseau, 2004). The correlation between key physical leaf and root traits remains controversial (Cheng et al., 2016). For example, the reported SLA–SRL relationship was positive (Withington et al., 2006), negative (Kembel and Cahill, 2011), or nonsignificant (Chen et al., 2013). The same holds for the correlation between leaf and fine-root nutrient contents (e.g., N and P), which was found to be positive in some studies (Tjoelker et al., 2005; Kerkhoff et al., 2006; Freschet et al., 2010; Holdaway et al., 2011), but not in others (Withington et al., 2006; Chen et al., 2013). These mixed findings suggest that the coordination between leaf and root traits may be contingent upon environmental conditions (e.g., abiotic stresses and soil properties), species composition, plant functional types (PFTs) and the spatial scale of interest (Geng et al., 2014; Cheng et al., 2016; Weemstra et al., 2016). In desert environments, leaf and fine-root traits are expected to be tightly coordinated because water and nutrient limitation to plant growth requires fine-root functions (i.e., water and nutrient uptake) to match those of leaves (i.e., photosynthesis and transpiration; Carvajal et al., 2019). We currently know little about the relative strength of stoichiometric vs. physical coordination between leaves and roots across desert plants.

Many leaf and root traits have been shown to differ among PFTs that are predefined by growth form (e.g., grass, forb, and woody species), taxonomy (e.g., monocot and eudicot) or functional categories (e.g., legumes, non-legumes; Freschet et al., 2017). Therefore, PFTs may be useful in categorizing species trait syndromes (Tjoelker et al., 2005; Caplan et al., 2019). For example, global analyses showed that graminoids had generally lower fine-root N content and tissue density than forbs, shrubs, and trees (Freschet et al., 2017). A local-scale study in a subarctic flora suggested that differences among growth forms can also be seen for structural traits such as SRL and RTD (Freschet et al., 2010). Although individual leaf and fine-root traits were observed to differ among PFTs, whether trait correlations differ among PFTs in predictable ways remains poorly understood (Tjoelker et al., 2005).

Current knowledge on plant functional traits is mainly obtained from studies on forests and grasslands (Craine et al., 2005; Kong et al., 2014; Cheng et al., 2016; Zhang et al., 2018). Species from the desert region should display strategies favouring higher belowground (water and nutrient) than aboveground (light) resource acquisition compared with species from forests or grasslands (Liu et al., 2010). However, it remains largely unclear whether existing theories are equally applicable to desert plants. Desert shrub communities constitute an ideal system to test the traits correlations because water is considered the main resource limiting plant abundance and distribution (Carvajal et al., 2019). We examined how leaf and fine-root traits are correlated across species and differ among PFTs in a desert shrubland. Specifically, we tested the hypotheses that (1) the leaf-trait syndrome of plant species in desert shrublands follows the predictions of the global LES, and is paralleled by a similar fine root-trait syndrome, (2) functional traits related to nutrient contents and resource uptake are tightly coordinated between leaves and fine roots in desert ecosystems where plant growth is limited primarily by dry and nutrient-poor conditions, and (3) traits as well as their relationships vary among PFTs.

## Materials and Methods

### Study Site

This study was conducted at the Yanchi Research Station (37°42′31″N, 107°13′37″E, 1530ma.s.l.), Ningxia, northern China. The site is located at the southern edge of the Mu Us Desert and is characterized by a temperate semiarid continental climate. The mean annual temperature (1954–2020) is 8.4°C, and the mean annual precipitation is 293mm. Most precipitation (>70%) occurs during June to September (data source: Yanchi Meteorological Station, Yanchi Research Station). The soil is a Arenosols (The FAO-UNESCO soil classification) with a total nitrogen content of 0.1–0.2gkg^−1^ and a soil organic carbon (C) content of about 2.0gkg^−1^. The landscape of this region is typical of inland dune ecosystems, which are colonized mainly by deciduous perennial shrubs, perennial grasses and annuals (She et al., 2017). The study area experienced severe desertification during the 1960–1990’s due to human disturbances (e.g., overgrazing and reclamation). Large-scale conservation practices (e.g., fencing and grazing ban) over the recent two decades have promoted the recovery of natural vegetation (Bai et al., 2018). The studied shrubland community is located in a conserved area in which human activities are negligible and all plants grow naturally. The shrubland community is dominated by a mixture of xerophytic shrub species, including *Artemisia ordosica*, *Hedysarum mongolicum*, and *Salix psammophila*. Most abundant herbaceous species include *Leymus secalinus*, *Stipa glareosa*, *Pennisetum centrasiaticum*, and *Setaria viridis*. Germination and leaf emergence usually start in mid-April, and the growing season ends in around mid-October. Leaf area index (LAI) at mid-growing season varies from year to year, and can exceed 1.0m^−2^ m^2^ in most productive years. LAI declines virtually to zero during winter as all plant species are cold-deciduous.

### Field Sampling and Trait Measurements

Four plots (40m×40m, 20m apart from each other) were set in the studied shrubland community in the spring of 2019. The four plots were considered replicates based on their similarity in topography and soil properties (Supplementary Table 1) as well as in species composition. The similarity in species composition was quantified with the Jaccard index (Qin et al., 2019), which ranged from 0.61 to 0.71. We then ranked all species in each plot by relative abundance and sampled all dominant species (i.e., relative abundance >5%). Following the standard trait collection protocols detailed in Cornelissen et al. (2003) and other studies (Liu et al., 2010; Geng et al., 2014; Mitchell et al., 2017), we sampled five mature individuals of each dominant species (to minimize labor and disturbance) from each plot. A total of 20 individuals (five ind.×four plots) were sampled for most species, while 10–15 individuals were sampled for those which occurred in only two or three plots. For each individual we collected five fully-expanded, fresh and healthy leaves and 10 fine roots (diameter<2mm; Cornelissen et al., 2003; Kong et al., 2014; Mitchell et al., 2017). This sampling strategy ensures that all field sampling and measurements can be done during the mid-growing season (from late June to late August), and that sampled species can represent the community assembly. We acknowledge that our small sample size for each species may not be adequate for examining intraspecific trait variations. However, the sampling method described here has been commonly used to investigate trait variations and relationships across species (Geng et al., 2014).

For shrub species, we carefully excavated the soil (0–30cm) at the base of each individual whose leaves had been collected, exposing the coarse roots. To ensure fine roots of the target individual were sampled, we followed each coarse root to find the attachment points of fine roots of the target plant. The points at which intact fine roots were attached to the coarse root were then determined using vernier calipers and cut with scissors. For herbaceous species, we carefully collected whole plants back to lab for the separation of leaves and fine roots. Active fine roots (generally have a lighter color and a fully turgid appearance) of each individual were identified according to root color, texture and connection to its shoot (Cheng et al., 2016).

A total of 10 shrub and 38 herbaceous species were investigated, covering 39 genera and 16 families. All sampled species are deciduous, including 33 perennials, three biennials, two annual grasses and 10 annual forbs (Supplementary Table 2). Leaf traits were measured for all 48 species (Supplementary Table 2; Supplementary Figure 1), while fine-root traits were measured for a subset of 43 species as fine roots were difficult to collect for five herbaceous species. We measured functional traits for both leaves and fine roots, including SLA, SRL, LTD, LN, RN, LP, root phosphorous content (RP), leaf and root organic C contents (LC and RC, respectively), and further calculated LN:LP, RN:RP, LC:LN, and RC:RN ratios.

Plant functional traits of sampled species were measured following standardized protocols detailed in Cornelissen et al. (2003). All samples were sealed in plastic bags, placed on ice, and returned to the lab where leaf samples were digitally imaged within 1h of collection. Leaf thickness was determined with electronic vernier calipers, and leaf area was measured using the Image J software.^1^ Leaf volume was calculated as the product of leaf thickness and leaf area. We carefully removed the remaining soil and organic matter from the fine-root samples using deionized water and tweezers. In the absence of a digital image analysis system, we measured the length of fine roots manually. Put the fine-root samples on the glass plate with grid paper and measure its length by straightening both ends with tweezers (Cheng et al., 2005). Leaf and fine-root samples were oven dried at 75°C for 48h to constant weight and weighed to calculated SLA (leaf area per unit dry mass, cm^2^ g^−1^), SRL (fine-root length per unit dry mass m g^−1^) and LTD (leaf dry mass per total volume, g cm^−3^). The C, N contents (gkg^−1^) of leaf and fine-root samples were measured through an elemental analyzer (Vario Max CN Element Analyser, Elementar, Germany) and total P content (gkg^−1^) was analyzed colorimetrically after H_2_SO_4_-H_2_O_2_-HF digestion (John, 1970).

### Statistical Analysis

All data were logarithmically transformed prior to analysis to satisfy the assumption of normality. The N:P and C:N ratios represent mass ratios in this study. Investigated plants were sorted into PFTs for analyzing differences in trait values and bivariate trait relationships among groups. Specifically, they were classified into grasses, forbs, and woody species based on life form, into legumes and non-legumes based on their ability to fix nitrogen, and into monocots and eudicots based on their evolutionary relationships. We did not compare deciduous vs. evergreen species because the latter are virtually absent from our study site due to cold winter. Nor did we compare perennial vs. annual species, as this classification largely confounds that based on life form (i.e., all woody species and most grasses are perennial). Species mean trait values were used for testing trait correlations, due to our focus on trait relationships across species. Bivariate trait relationships were tested with a model II (standardized major axis, SMA) regression, which is commonly used when independent variable is not clearly defined and/or measurement errors exist for both variables (Craine et al., 2005). SMA slopes and y-intercepts were calculated using the “smatr” package of the R software. Due to multiple trait correlations, a principal component analysis (PCA) was performed for all leaf and fine-root traits (“whole-plant PCA” hereafter) to test overall patterns of trait variations (Craine et al., 2005). All variables used in PCA were standardized to a mean of zero and a SD of one. A separate PCA was also performed for all leaf traits (“leaf PCA” hereafter) or all fine-root traits (“root PCA” hereafter), to examine overall trait variations in leaves and fine roots, respectively. Differences in any leaf or fine-root traits among PFTs and species were tested using a nested ANOVA (nested ANOVA), in which functional type was treated as a fixed factor, and species was treated as a random factor nested within functional type. The Tukey HSD method was used for *post hoc* multiple comparisons. Multivariate analysis of variance (MANOVA) was performed to test whether species scores on the first two PCA axes show overall differences among grasses, forbs, and woody species, and Hotelling’s *T*^2^-test was performed instead when comparing between legumes and non-legumes, or between monocots and eudicots. In addition, ANOVA and *t*-test were performed on species scores to compare PFTs along individual PCA axes.

To remove the effects of phylogenetic relatedness among species (due to shared evolutionary history) on trait variations, we calculated phylogenetically independent contrasts (PIC, Felsenstein, 1985) using the “ape” R package to further evaluate pairwise correlations between leaf and fine-root traits (Kerkhoff et al., 2006; Geng et al., 2014). Investigated species were sorted into genera and families based on the APG III classification using the “plantlist” R package (The Angiosperm Phylogeny Group, 2009), and a supertree for all taxa was built using the freely available software Phylomatic.^2^ Because PICs were calculated based on nonnegative *x*-axis contrasts, we forced the SMA regressions on PICs through the origin following Kerkhoff et al. (2006). All statistical analyses were conducted in R version 4.0.3 (The R development Core Team). The significance level was set as *p*=0.05.

## Results

### Trait Correlations in Leaves and Fine Roots Across All Species

**Figure 1:**
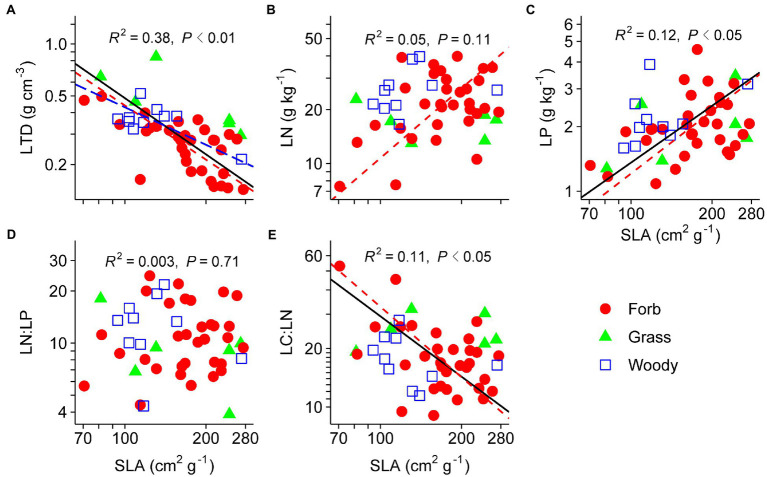
Relationships between specific leaf area (SLA) and leaf tissue density (LTD; **A**), leaf nitrogen content (LN; **B**), leaf phosphorus content (LP; **C**), leaf N:P ratio (LN:LP; **D**), and leaf C:N ratio (LC:LN; **E**). Species means are shown for forbs (closed circle), grasses (triangle), and woody species (open square). Solid lines (*R*^2^ and *p* values) represent linear fits across all species, red dashed lines represent linear fits for forbs, and blue long-dashed lines represent linear fits for woody species. Type II model was used for all linear fits. The log_10_ scale was used on both *x*- and *y*-axis.

**Figure 2:**
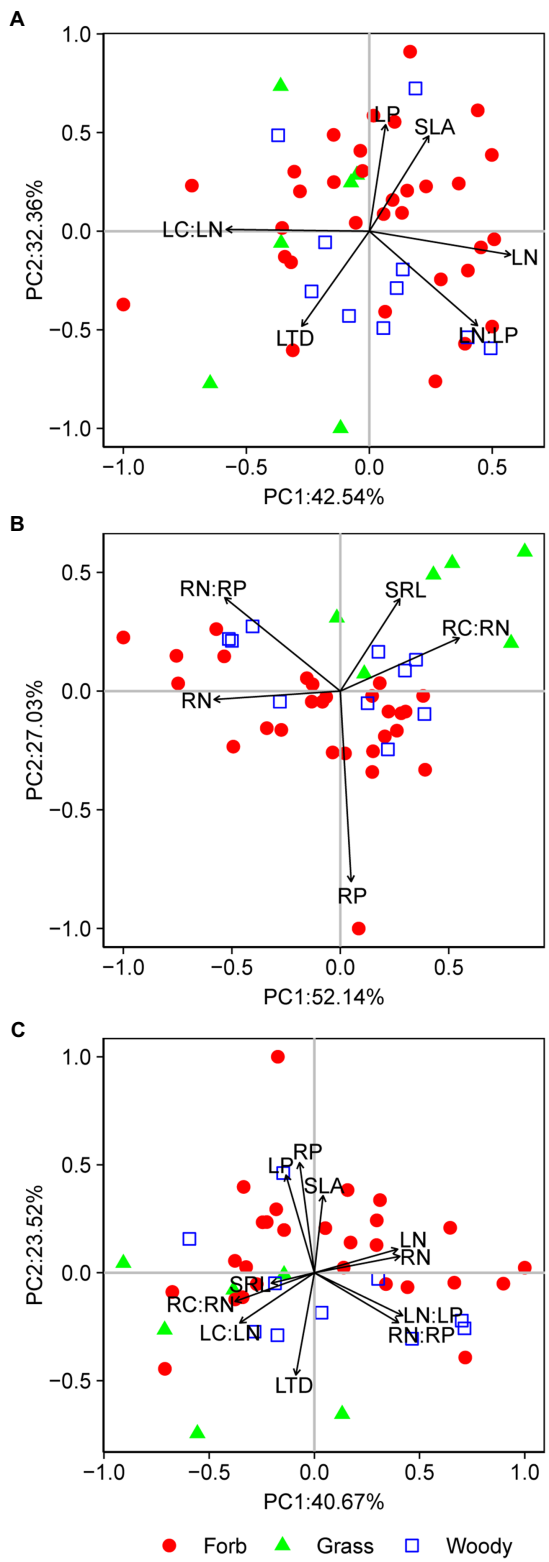
Principal component analyses (PCAs) for leaf traits **(A)**, fine-root traits **(B)**, and whole-plant traits **(C)**. Each data point represents the position of a forb (closed circle), grass (triangle), or woody species (open square) in the two-dimensional trait space. SLA, specific leaf area; LTD, leaf tissue density; LN, leaf nitrogen content; LP, leaf phosphorus content; LN:LP, leaf N:P ratio; LC:LN, leaf C:N ratio; SRL, specific root length; RN, fine-root nitrogen content; RP, fine-root phosphorus content; RN:RP, fine-root N:P ratio; and RC:RN, fine-root C:N ratio. The percentages on *x*- and *y*-axis indicate the amount of variance explained by the two main axes.

Pairwise trait relationships revealed that SLA was positively correlated with LP, negative correlated with LTD and LC:LN, but not correlated with LN and LN:LP across all species (Figure 1). Surprisingly, LN and LP were not correlated (*p*=0.21). The first two main axes (PC1 and PC2) for the leaf PCA explained 43 and 32% of total variance, respectively, in selected leaf traits. Leaf PCA was generally consistent with pairwise relationships, with PC1 showing that species with low SLA had high LTD and LC:LN, but low LN, LP, and LN:LP (Figure 2A and Table 1). SRL was independent of other fine-root traits (Supplementary Figure 2). PC1 and PC2 for the root PCA explained 52 and 27% of total variance, respectively, in examined fine-root traits. PC1 for the root PCA showed that species with low SRL generally had high RN, and RN:RP and low RP and RC:RN (Figure 2B and Table 1).

**Table 1:** Coefficients for eigenvectors for main axes of principal component analyses (PCAs) on leaf and/or fine-root traits.

Traits	Leaf or root PC1	Leaf or root PC2	Whole-plant PC1	Whole-plant PC2
SLA	0.24	0.48	0.04	0.36
LTD	−0.27	−0.48	−0.09	−0.47
LN	0.57	−0.12	0.40	0.11
LP	0.07	0.54	−0.13	0.45
LN:LP	0.44	−0.48	0.42	−0.20
LC:LN	−0.58	0.01	−0.35	−0.23
SRL	0.27	0.39	−0.20	−0.05
RN	−0.58	−0.04	0.41	0.08
RP	0.05	−0.80	−0.07	0.51
RN:RP	−0.53	0.39	0.40	−0.23
RC:RN	0.55	0.22	−0.38	−0.13

SLA, specific leaf area; LTD, leaf tissue density; LN, leaf nitrogen content; LP, leaf phosphorus content; LN:LP, leaf N:P ratio; LC:LN, leaf C:N ratio; SRL, specific root length; RN, fine-root nitrogen content; RP, fine-root phosphorus content; RN:RP, fine-root N:P ratio; and RC:RN, fine-root C:N ratio.

### Correlations Between Leaf and Fine-Root Traits Across All Species

**Figure 3:**
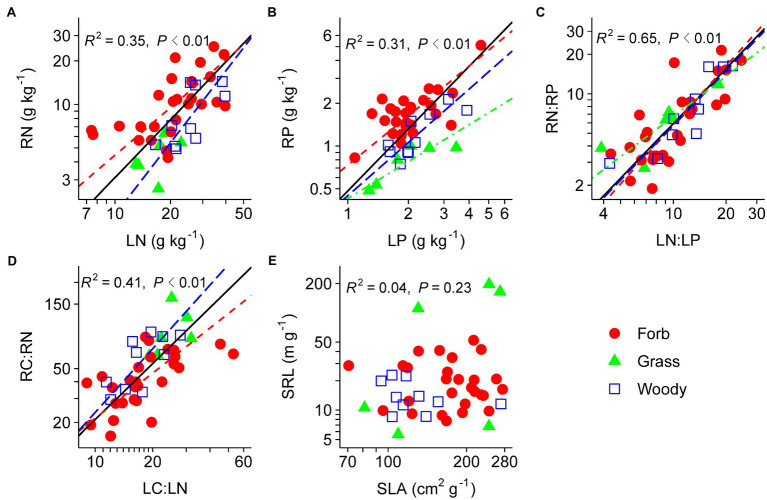
Relationships between analogous leaf and fine-root traits across species, including the RN–LN relationship **(A)**, the RP–LP relationship **(B)**, the RN:RP–LN:LP relationship **(C)**, the RC:RN–LC:LN relationship **(D)**, and the SRL–SLA relationship **(E)**. For abbreviations see Figures 1, 2. Species means are shown for forbs (closed circle), grasses (triangle), and woody species (open square). Solid lines (*R*^2^ and *p* values) represent linear fits across all species, red dashed lines represent linear fits for forbs, blue long-dashed lines represent linear fits for woody species, and green dot-dashed lines represent linear fits for grasses. Type II model was used for all linear fits. The log_10_ scale was used on both *x*- and *y*-axis.

Nutrient-related traits (N, P, N:P, and C:N) were all positively correlated between leaves and fine roots (Figures 3A–D). SLA and SRL were unrelated (Figure 3E). After controlling for phylogenetic relatedness among species, N and P contents, and N:P ratio were tightly coordinated between leaves and fine roots, whereas the correlations between SRL and SLA and between LC:LN and RC:RN were marginally significant (Table 2).

**Table 2:** Phylogenetically-independent contrasts between leaf and fine-root traits among desert species.

Root vs. leaf trait	*R* ^2^	*p*	*n*
logSRL vs. logSLA	0.47	0.05	8
logRN vs. logLN	0.45	<0.01	16
logRP vs. logLP	0.86	<0.01	11
log(RN:RP) vs. log(LN:LP)	0.59	<0.01	24
log(RC:RN) vs. log(LC:LN)	0.30	0.06	11

For abbreviations see Table 1.

The whole-plant PCA revealed two independent sets of correlations (Figure 2C). PC1 accounted for 41% of total variance in leaf and fine-root traits, compared to 24% explained by PC2 (Figure 2C and Table 1). PC1 represented a continuous distribution of species from those that have low N, N:P ratio and high C:N ratio leaves and fine roots to those that have high N, N:P ratio and low C:N ratio leaves and fine roots (Figure 2C and Table 1). PC2 represented a continuum of species from those characterized by high SLA and tissue P but low LTD to those with low SLA and tissue P but high LTD. Moreover, plant scores on the first two axes of the root PCA were, respectively, correlated with their scores on the first two axes of the leaf PCA (for PC1: *R*^2^=0.44, *p*<0.01; for PC2: *R*^2^=0.26, *p*<0.01).

### Variations in Leaf and Fine-Root Traits Among PFTs

SRL was higher in monocots than in eudicots, and highest in grasses and lowest in woody species (Supplementary Figure 3A). LTD was higher in monocots than in eudicots, and highest in grasses and lowest in forbs (Supplementary Figure 3B). Legumes had generally higher N and lower P (therefore higher N:P and lower C:N) than non-legumes (Supplementary Figure 4). RN and RP were higher in eudicots than in monocots, and highest in forbs and lowest in grasses (Supplementary Figures 4A,B). RC:RN ratio was higher in monocots than in eudicots, and highest in grasses and lowest in forbs (Supplementary Figure 4D).

Significant correlations between SLA and LTD were found in all PFTs except for monocots and grasses (Table 3). SLA was positively correlated with LN and LP in forbs and eudicots, and was negatively correlated with LC:LN in forbs, eudicots, and non-legumes. LP also increased with SLA in non-legumes. N and P contents were significantly correlated in leaves (*R*^2^=0.22, *p*<0.01) and fine roots (*R*^2^=0.24, *p*<0.01) of non-legumes, but not in other PFTs. SRL was largely uncorrelated with other fine-root traits in individual PFTs (Supplementary Table 3), exceptions were RN–SRL (positive) and RC:RN–SRL (negative) relationships in legumes, and RP–SRL (positive) and RN:RP–SRL (negative) relationships in eudicots.

**Table 3:** Relationships between SLA (*x*) and other leaf traits (*y*) for different functional types (PFTs).

*y*	PFT	*a*	*b*	*R* ^2^
logLTD	Forb (32)	1.73	−1.04^***^	0.41
	Woody (10)	1.08	−0.72^*^	0.43
	Eudicot (42)	1.69	−1.02^***^	0.47
	Legume (13)	1.31	−0.82^*^	0.31
	Non-legume (35)	1.92	−1.12^***^	0.41
logLN	Forb (32)	−1.56	1.29^**^	0.20
	Eudicot (42)	−1.25	1.18^*^	0.10
logLP	Forb (32)	−1.86	0.97^*^	0.15
	Eudicot (42)	−1.69	0.91^*^	0.11
	Non-legume (35)	−1.50	0.84^**^	0.19
log(LC:LN)	Forb (32)	3.92	−1.20^**^	0.22
	Eudicot (42)	3.63	−1.09^**^	0.17
	Non-legume (35)	3.37	−0.94^*^	0.15

For abbreviations see Table 1. Symbols *a* and *b* represent the intercept and slope of linear regression, respectively, *R*^2^ represents the coefficient of determination. Only significant relationships are shown. Species numbers are included in parentheses.

* Indicates significant linear regression at 0.05 confidence levels.

** Indicates significant linear regression at 0.01 confidence levels.

*** Indicates significant linear regression at 0.001 confidence levels.

None of the PFTs showed significant correlation between SLA and SRL (Figure 3E, other results not shown). Fine-root nutrient contents increased with leaf nutrient contents in forbs, woody species, eudicots, and non-legumes (Figure 3 and Table 4). LN and LC:LN were significantly correlated with RN and RC:RN, respectively, in eudicots but not monocots.

**Table 4:** Correlations between leaf and fine-root traits for different functional types (PFTs).

*y*	*x*	PFT	*a*	*b*	*R* ^2^
logRN	logLN	Forb (27)	−0.45	1.10^***^	0.39
		Woody (10)	−1.42	1.65^*^	0.50
		Eudicot (37)	−0.59	1.17^***^	0.32
		Non-legume (32)	−0.47	1.01^*^	0.17
logRP	logLP	Grass (6)	−0.37	0.86^*^	0.75
		Forb (27)	−0.14	1.16^***^	0.44
		Woody (10)	−0.35	1.25^**^	0.67
		Monocot (6)	−0.37	0.86^*^	0.75
		Eudicot (37)	−0.23	1.28^***^	0.35
		Non-legume (32)	−0.34	1.48^***^	0.35
log(RN:RP)	log(LN:LP)	Grass (6)	−0.19	1.02^*^	0.68
		Forb (27)	−0.73	1.50^***^	0.62
		Woody (10)	−0.63	1.39^***^	0.78
		Monocot (6)	−0.19	1.02^*^	0.68
		Eudicot (37)	−0.70	1.46^***^	0.67
		Non-legume (32)	−0.44	1.18^***^	0.46
log(RC:RN)	log(LC:LN)	Forb (27)	0.22	1.11^***^	0.40
		Woody (10)	−0.19	1.57^*^	0.52
		Eudicot (37)	0.10	1.24^***^	0.36
		Non-legume (32)	0.39	1.06^**^	0.27

For abbreviations see Table 1. Symbols *a* and *b* represent the intercept and slope of linear regression, respectively, *R*^2^ represents the coefficient of determination. Only significant relationships are shown. Species numbers are included in parentheses.

* Indicates significant linear regression at 0.05 confidence levels.

** Indicates significant linear regression at 0.01 confidence levels.

*** Indicates significant linear regression at 0.001 confidence levels.

MANOVA and Hotelling’s *T*^2^-test revealed that species scores on the first two main axes (PC1 and PC2) of leaf, root, and whole-plant PCA generally differ among PFTs, except for those for leaf PCA among grasses, forbs, and woody species and between monocots and eudicots (Supplementary Table 4). ANOVA and *t*-test for PC1 showed significant functional type effects on species scores except for leaf traits among grasses, forbs, and woody species; and ANOVA and *t*-test for PC2 also generally supported trait differentiation among PFTs, albeit with a few expectations (Supplementary Table 5).

## Discussion

### Do Leaf Traits Conform to the LES Theory?

The LES theory predicts that species with high SLA are characterized by low LTD, high mass-based nutrient contents, high photosynthetic and respiration rates, and short life span, while species with low SLA usually show the opposite pattern of leaf traits (Wright et al., 2004). Our results are partially consistent with our first hypothesis that leaf traits of desert shrubland species follow the LES theory. The correlation between SLA and LN, as predicted by LES, was not supported in this study. Positive correlations between SLA and LN have been widely reported in previous leaf trait studies (Reich, 2014), with the exception of Zhou et al. (2010), who found that SLA and LN were decoupled across dominant species of the Inner Mongolia grassland. The decoupling between LN and LP we found is against the ecological stoichiometry theory (Zhang et al., 2018), but similar to the finding from a semi-desert community (Grubb et al., 2015).

Such a pattern among leaf traits implies that LN is not necessarily related to the acquisitive strategy in harsh environments (e.g., drylands; He et al., 2008). First, desert species may store part of absorbed N in leaves when plant growth is strongly water-limited (Zhou et al., 2010). Stored N does not contribute directly to the “fast” syndrome. Second, LN is not only involved in photosynthesis, but also comprises compounds that play important non-photosynthetic roles (e.g., defense against herbivory and energy production for metabolism; Osnas et al., 2013). Therefore, the multiple functions of LN (e.g., photosynthesis, storage, and defense) should be explicitly considered in investigating trait variations, trade-offs, and plant strategies in desert communities.

### Is There a Root-Trait Syndrome in Parallel With the Leaf-Trait Syndrome?

The RES assumes that leaf traits are matched by parallel root traits along the acquisitive-conservative resource spectrum (Reich, 2014; Weemstra et al., 2016), and the theory predicts that plant roots with high SRL are also characterized by low RTD but high nutrient contents, turnover rates, and respiration rates. However, the existence of an RES analogous to the LES is currently debated, and evidence has been mixed among and within studies (Withington et al., 2006; Kong et al., 2014; Weemstra et al., 2016). Our results showed generally weak or no correlation between fine-root traits, and thus do not support our hypothesis that the leaf-trait syndrome is paralleled by a similar root-trait syndrome. Similar to our results, some studies in forests and grasslands also found a lack of correlation between SRL and RN (Tjoelker et al., 2005; Chen et al., 2013; Weemstra et al., 2016) and between SRL and RTD (Craine et al., 2001; Chen et al., 2013; Kramer-Walter et al., 2016).

Several reasons may account for the lack of a root-trait syndrome that parallels the leaf-trait syndrome. With regard to methodology, the sampled fine roots (<2mm in diameter) were not necessarily all absorptive roots. The possible inclusion of fine transport roots (second- or higher-order roots), which do not represent resource uptake strategies, may confound trait relationships (Withington et al., 2006; McCormack et al., 2015). Root order rather than diameter may be a better proxy for root functioning (McCormack et al., 2015). Therefore, our conclusion of a lack of fine-root trait syndrome is tentative and should be verified in future studies with functional root classifications or order-based analyses. With regard to ecological mechanisms, roots are subjected to multiple constraints especially in desert ecosystems (e.g., uptake of water and multiple nutrients), while leaves are mainly adapted for maximizing carbon gain during their lifetimes (Weemstra et al., 2016). Soil physical and chemical properties in desert regions (such as density, pH, and cation exchange capacity) may present additional limits to root traits that are not present aboveground. In addition, leaf and root traits are not necessarily analogous, because they function differently and might not be related to resource uptake in a similar manner (Weemstra et al., 2016). Finally, resource acquisition by roots is strongly influenced by their interactions with mycorrhizal fungi and other rhizospheric organisms, resulting in selection forces on root traits that are distinct from those on leaf traits (Withington et al., 2006; Reich, 2014). Therefore, a lack of root-trait syndrome that parallels the leaf-trait syndrome across desert shrubland species implies that a multidimensional root trait framework (Weemstra et al., 2016) that incorporates multiple root functions, multiple constraints on root traits and plant-mycorrhizal interactions may be developed for understanding root-trait variations and correlations in deserts and other stressful environments.

### Are Fine-Root Traits Coordinated With Analogous Leaf Traits?

Our results revealed that plant nutrient contents rather than physical traits (i.e., SLA and SRL) were coordinated between leaves and fine roots in the studied shrubland, a pattern partially in line with our second hypothesis. The whole-plant economics spectrum assumes SRL to be analogous to SLA (Reich, 2014), as these two traits represent resource acquisition capability by fine roots and leaves, respectively. However, available evidence for the linkage between SRL and SLA is mixed at best (Withington et al., 2006). Some studies revealed positive SLA–SRL relationships in woody (Withington et al., 2006; Holdaway et al., 2011) and herbaceous species (Cheng et al., 2016), while other studies found either negative or a lack of correlation between SLA and SRL in different regions and ecosystem types (Kembel and Cahill, 2011; Chen et al., 2013; Geng et al., 2014). Our finding of the nonsignificant SRL–SLA relationship also suggest that SRL may not be the functional analogue of SLA in desert shrublands. In contrast to leaves, the link between root physical traits and resource uptake are not well-established, and SRL might not be a adequate predictor of belowground resource acquisition capacity in desert ecosystems (Eissenstat et al., 2000; Weemstra et al., 2016). Firstly, root functioning (e.g., absorptive vs. transport) is strongly affected by its branching order, even for herbaceous species which do not have as many root branches and complex structures as do woody plants. This may lead to differences in root and leaf physical traits, obscuring the SRL–SLA relationship (Geng et al., 2014; Cheng et al., 2016). In addition, the fine roots of herbaceous species in this study may have a relatively small range of variation in trait values, and as a consequence, the SLA–SRL relationship is likely to be nonsignificant (Geng et al., 2014). Secondly, many desert plants rely on mycorrhizal hyphae to efficiently exploit the soil, and small SRL may support more mycorrhizal fungal colonization per unit root length (Comas et al., 2002; McCormack et al., 2015). Thirdly, root physical traits such as diameter and SRL may be more phylogenetically conservative than leaf physical traits, leading to weak physical coordination between organs (Comas and Eissenstat, 2009; Chen et al., 2013). Therefore, explicit consideration of fine-root functions and assessment of plant-mycorrhizal interactions may aid in the understanding of leaf-root coordination in desert plants.

Plant nutrient contents were significantly positively correlated between leaves and fine roots (Figures 2C, 3). These results are in line with previous studies on temperate grasslands (Craine et al., 2005) and forests (Holdaway et al., 2011). Furthermore, the PICs confirmed that the nutrient coordination between leaves and fine roots were not the result of phylogenetic relatedness among studied species (Table 2). Such a nutrient-based leaf-root coordination thus reflects the consistency in nutrient uptake and allocation above- and below-ground in desert shrublands. The tight coordination between leaf and root nutrient contents provides the potential to predict belowground stoichiometry from aboveground measurements.

### Do Functional Types Summarize Differences in Traits and Trait Relationships?

Large uncertainties exist on the extent to which trait syndromes are able to differentiate among predefined PFTs (Cheng et al., 2016; Verheijen et al., 2016). Our results support our third hypothesis, showing that PFTs summarized a significantamount of variability in plant traits. Our finding at the local scale is consistent with a recent global synthesis (Verheijen et al., 2016), which demonstrated that PFTs of desert community were differently positioned in the multidimensional trait space. Similarly, Craine et al. (2001) found that grasses and forbs in central Minnesota prairies had distinct trait syndromes. In contrast, Cheng et al. (2016) showed that two key traits, SLA and SRL, were capable of classifying 55 species in the Inner Mongolia grassland into phylogenetically different groups (i.e., early diverged species vs. late diverged species), rather than into distinct PFTs.

Despite the potential of plant traits in discriminating among PFTs, both our results and previous studies reported large variations within PFTs and overlaps between PFTs in plant strategies and traits (Van Bodegom et al., 2012; Verheijen et al., 2013). This indicates that a wide range of strategies may be used by plants within a single PFT to adapt to similar environment conditions, and that some plants may show traits similar to that of other PFTs (Craine et al., 2001). For example, in the studied desert community some grasses such as *Pennisetum centrasiaticum* and *Leymus secalinus* were more like forbs in the whole-plant trait space, while some forbs such as *Corispermum hyssopifolium* and *Bassia dasyphylla* had leaves and roots traits that resemble grasses (Figure 2C). We propose that one of the future research challenges in trait-based ecology is to understand what determines the potential of plant traits to functionally differentiate among PFTs, as this potential would allow global or regional vegetation mapping based on trait maps (Van Bodegom et al., 2014; Verheijen et al., 2016).

The ability of plant traits to differentiate among PFTs depends partly on how PFTs are classified. Leaf and roots traits in our study best discriminated between legumes and non-legumes. Another source of uncertainty in differentiating among PFTs is the selection of trait combinations (Verheijen et al., 2016). Differentiations between growth forms (grasses, forbs, and woody species) or evolutionary relationships (monocots and eudicots) were mostly attributed to root rather than leaf traits. Despite the importance of root traits in differentiating among PFTs, variations in some root traits among PFTs are not in line with the presumed RES. For example, low RN and RP in grasses exhibiting high SRL (Supplementary Figures 3, 4), indicating that their roots have relatively low metabolic rates and depend mainly on cost-efficient root structure to acquire soil resources (Reich et al., 2008; Freschet et al., 2017). Future studies should examine which traits and classifications are most relevant to functional differences among PFTs. Incorporation of trait variations among the most relevant classification of PFTs should improve the modelling of plant and ecosystem functioning.

In line with our third hypothesis, PFTs also differed in bivariate relationships between leaf and fine-root traits. Most trait correlations did not hold in all PFTs, suggesting different nutrient absorption and utilization characteristics among PFTs. Therefore, the influence of PFTs on trait associations and trade-offs should be considered when estimating one trait from another. In addition, differences in bivariate trait relationships among PFTs could provide important insights into the mechanisms governing species effects on ecosystem processes (Tjoelker et al., 2005).

## Conclusions

Our analyses using 48 species in a desert shrubland community of northern China revealed that variations in leaf traits were partially in line with the predictions of the global LES. Variations in fine-root traits, however, provided little evidence for a RES. The coordination between leaves and fine roots was stronger for nutrient contents and their ratios than for physical traits (i.e., SLA and SRL). In addition, our results illustrate the potential of plant traits to functionally differentiate among PFTs, despite large overlaps among PFTs in plant strategies. We conclude that fine-root functions in the studied desert community are probably coordinated with leaf functions with respect to nutrient allocation and use. Future studies at the regional scale should examine the extent to which our conclusions are applicable across all types of desert communities.

## Data Availability Statement

Data used in this study are available as part of the Supplementary Material.

## Author Contributions

XinJ designed and led this research. XiaJ and XinJ wrote the draft manuscript and analyzed the data. XiaJ, SG, YJ, NW, and CH performed field sampling and measurements. TZ, PL, YT, and SQ provided editorial advices. All authors contributed to the article and approved the submitted version.

## Funding

This study was supported by the National Natural Science Foundation of China (NSFC, nos. 32071843, 31670708, 31901366, and 32071842) and the Fundamental Research Funds for the Central Universities (nos. 2015ZCQ-SB-02, PTYX202122, and PTYX202123).

## Conflict of Interest

The authors declare that the research was conducted in the absence of any commercial or financial relationships that could be construed as a potential conflict of interest.

## Publisher’s Note

All claims expressed in this article are solely those of the authors and do not necessarily represent those of their affiliated organizations, or those of the publisher, the editors and the reviewers. Any product that may be evaluated in this article, or claim that may be made by its manufacturer, is not guaranteed or endorsed by the publisher.

## Footnotes

^1^ https://imagej.net/Welcome

^2^ http://www.phylodiversity.net/phylomatic
