# Supplementary material for: Plant Nutrient Contents Rather Than Physical Traits Are Coordinated Between Leaves and Roots in a Desert Shrubland
Source: Front Plant Sci. 2021 Oct 26;12:734775. doi: 10.3389/fpls.2021.734775 (PMC8576145; doi:10.3389/fpls.2021.734775)
Supplement: Supplementary file 1 [file Data_Sheet_1.docx]

Supplementary Material

**Supplementary Table 1.** Topography and soil properties in the four studied plots (Mean±SE)^a^.

| Variable | plot 1 | plot 2 | plot 3 | plot 4 |
| --- | --- | --- | --- | --- |
| Slope (°) | 14.25±2.72^a^ | 9.50±7.51^a^ | 9.75±1.80^a^ | 6.00±2.80^a^ |
| Soil organic carbon (C, g kg^-1^) | 0.92±0.02^a^ | 2.48±0.48^b^ | 2.37±0.22^b^ | 1.96±0.36^ab^ |
| Soil total phosphorus (P, g kg^-1^) | 0.22±0.01^a^ | 0.30±0.03^a^ | 0.28±0.02^a^ | 0.29±0.02^a^ |
| Soil total nitrogen (N, g kg^-1^) | 0.05±0.01^a^ | 0.16±0.07^a^ | 0.15±0.02^a^ | 0.15±0.03^a^ |
| Soil N:P | 0.22±0.02^a^ | 0.47±0.15^a^ | 0.52±0.07^a^ | 0.50±0.10^a^ |
| Soil C:N | 21.97±2.17^a^ | 21.08±3.42^a^ | 17.04±1.25^a^ | 14.79±1.13^a^ |

^a^ Soil was sampled soils at 0-30 cm depth at four corners of each plot. The soil samples were sieved through a 2-mm mesh and air-dried for soil organic carbon (SOC), and total nitrogen (TN) and phosphorus (TP) measurements. The C, N contents (g kg^-1^) of soil samples were measured through an elemental analyzer (Vario Max CN Element Analyser, Elementar, Germany) and total P content (g kg^-1^) was analyzed colorimetrically after H_2_SO_4_-H_2_O_2_-HF digestion.

**Supplementary Table 2.** Botanical information of each species.

| Species | Family | Life form | Evolutionary relationships | Life history | Growth  form |
| --- | --- | --- | --- | --- | --- |
| *Chloris virgata* | Gramineae | Grass | Monocot | Annual | Deciduous |
| *Pennisetum centrasiaticum* | Gramineae | Grass | Monocot | Perennial | Deciduous |
| *Phragmites australis* | Gramineae | Grass | Monocot | Perennial | Deciduous |
| *Leymus secalinus* | Gramineae | Grass | Monocot | Perennial | Deciduous |
| *Setaria viridis* | Gramineae | Grass | Monocot | Annual | Deciduous |
| *Stipa glareosa* | Gramineae | Grass | Monocot | Perennial | Deciduous |
| *Thermopsis lanceolata* | Leguminosae | Forb | Eudicot | Perennial | Deciduous |
| *Oxytropis racemosa* | Leguminosae | Forb | Eudicot | Perennial | Deciduous |
| *Astragalus melilotoides* | Leguminosae | Forb | Eudicot | Perennial | Deciduous |
| *Astragalus galactites* | Leguminosae | Forb | Eudicot | Perennial | Deciduous |
| *Fabaceae medicago* | Leguminosae | Forb | Eudicot | Perennial | Deciduous |
| *Astragalus adsurgens* | Leguminosae | Forb | Eudicot | Perennial | Deciduous |
| *Astragalus dahuricus* | Leguminosae | Forb | Eudicot | Annual | Deciduous |
| *Agriophyllum squarrosum* | Chenopodiaceae | Forb | Eudicot | Annual | Deciduous |
| *Corispermum hyssopifolium* | Chenopodiaceae | Forb | Eudicot | Annual | Deciduous |
| *Euphorbia esula* | Euphorbiaceae | Forb | Eudicot | Perennial | Deciduous |
| *Inula salsoloides* | Compositae | Forb | Eudicot | Perennial | Deciduous |
| *Silene aprica* | Caryophyllaceae | Forb | Eudicot | Annual | Deciduous |
| *Linum stelleroides* | Linaceae | Forb | Eudicot | Annual | Deciduous |
| *Cynanchum thesioides* | Asclepiadaceae | Forb | Eudicot | Perennial | Deciduous |
| *Chenopodium aristatum* | Chenopodiaceae | Forb | Eudicot | Annual | Deciduous |
| *Bassia dasyphylla* | Chenopodiaceae | Forb | Eudicot | Annual | Deciduous |
| *Euphorbia humifusa* | Euphorbiaceae | Forb | Eudicot | Annual | Deciduous |
| *Cynanchum chinense* | Asclepiadaceae | Forb | Eudicot | Perennial | Deciduous |
| *Incarvillea sinensis* | Bignoniaceae | Forb | Eudicot | Annual | Deciduous |
| *Mulgedium tataricum* | Compositae | Forb | Eudicot | Perennial | Deciduous |
| *Heteropappus altaicus* | Compositae | Forb | Eudicot | Perennial | Deciduous |
| *Sonchus arvensis* | Compositae | Forb | Eudicot | Perennial | Deciduous |
| *Ixeridium gracile* | Compositae | Forb | Eudicot | Perennial | Deciduous |
| *Inula japonica* | Compositae | Forb | Eudicot | Perennial | Deciduous |
| *Messerschmidia sibirica* | Boraginaceae | Forb | Eudicot | Perennial | Deciduous |
| *Chenopodium glaucum* | Chenopodiaceae | Forb | Eudicot | Annual | Deciduous |
| *Saussurea japonic* | Compositae | Forb | Eudicot | Biennial | Deciduous |
| *Melilotus officinalis* | Leguminosae | Forb | Eudicot | Biennial | Deciduous |
| *Plantago asiatica* | Plantaginaceae | Forb | Eudicot | Perennial | Deciduous |
| *Malva rotundifolia* | Malvaceae | Forb | Eudicot | Perennial | Deciduous |
| *Glycyrrhiza uralensis* | Papilionaceae | Forb | Eudicot | Perennial | Deciduous |
| *Onopordum acanthium* | Compositae | Forb | Eudicot | Biennial | Deciduous |
| *Lespedeza davurica* | Leguminosae | Woody | Eudicot | Perennial | Deciduous |
| *Caragana korshinskii* | Leguminosae | Woody | Eudicot | Perennial | Deciduous |
| *Hedysarum mongolicum* | Papilionaceae | Woody | Eudicot | Perennial | Deciduous |
| *Sophora alopecuroides* | Leguminosae | Woody | Eudicot | Perennial | Deciduous |
| *Periploca sepium* | Asclepiadaceae | Woody | Eudicot | Perennial | Deciduous |
| *Cynanchum komarovii* | Asclepiadaceae | Woody | Eudicot | Perennial | Deciduous |
| *Atraphaxis frutescens* | Polygonaceae | Woody | Eudicot | Perennial | Deciduous |
| *Salix cheilophila* | Salicaceae | Woody | Eudicot | Perennial | Deciduous |
| *Caryopteris mongholica* | Verbenaceae | Woody | Eudicot | Perennial | Deciduous |
| *Artemisia ordosica* | Compositae | Woody | Eudicot | Perennial | Deciduous |

**Supplementary Table 3.** Relationships between SRL (*x*) and other fine-root traits (*y*) for different functional types (PFTs).

| *y* | PFT | *a* | *b* | *R^2^* |
| --- | --- | --- | --- | --- |
| logRN | Legume (11) | 0.45 | 0.71** | 0.58 |
| logRP | Eudicot (37) | -0.72 | 0.74* | 0.13 |
| log(RN:RP) | Eudicot (37) | 2.38 | -1.29* | 0.11 |
| log(RC:RN) | Legume (11) | 2.32 | -0.84** | 0.55 |

For abbreviations see Table 1. Symbols *a* and *b* represent the intercept and slope of linear regression, respectively, *R^2^* represents the coefficient of determination. Only significant relationships are shown, with *, **, and *** indicating significant linear regression at 0.05, 0.01, and 0.001 confidence levels, respectively. Species numbers are included in parentheses.

**Supplementary Table 4.** Multivariate analysis of variance (MANOVA) and Hotelling’s *T*^2^-test using species scores on the first two main axes of leaf, root, or whole-plant principal component analysis (PCA).

| PFTs | Leaf PCA axes | | Root PCA axes | | Whole-plant PCA | |
| --- | --- | --- | --- | --- | --- | --- |
|  | *F* or *T*^2^ | *P* | *F* or *T*^2^ | *P* | *F* or *T*^2^ | *P* |
| Grass/forb/woody | 1.74 (4,90) | 0.15 | 8.07 (4,80) | <0.01 | 4.30 (4,80) | <0.01 |
| Legume/non-legume | 20.30 (2,45) | <0.01 | 47.62 (2,40) | <0.01 | 36.18 (2,40) | <0.01 |
| Monocot/eudicot | 2.40 (2,45) | 0.10 | 20.02 (2,40) | <0.01 | 7.78 (2,40) | <0.01 |

Numbers in parentheses represent numerator and denominator degrees of freedom.

**Supplementary Table 5.** Analysis of variance (ANOVA) and *t*-test using species scores on the first two main axes (PC1 and PC2, respectively) of leaf, root, or whole-plant principal component analysis (PCA).

| PFTs | Leaf PC1 | | Leaf PC2 | | Root PC1 | | Root PC2 | | Whole-plant PC1 | | Whole-plant PC2 | |
| --- | --- | --- | --- | --- | --- | --- | --- | --- | --- | --- | --- | --- |
|  | *F* or *t* | *P* | *F* or *t* | *P* | *F* or *t* | *P* | *F* or *t* | *P* | *F* or *t* | *P* | *F* or *t* | *P* |
| Grass/forb/woody | 2.23  (2,45) | 0.12 | 1.28  (2,45) | 0.29 | 5.16  (2,40) | 0.01 | 11.75  (2,40) | <0.01 | 3.19  (2,40) | 0.05 | 5.52  (2,40) | <0.01 |
| Legume/non-legume | 4.12 (46) | <0.01 | -3.44 (46) | <0.01 | -8.88 (41) | <0.01 | 1.41 (41) | 0.17 | 8.05 (41) | <0.01 | -1.16 (41) | 0.25 |
| Monocot/eudicot | -2.13 (46) | 0.04 | -0.55 (46) | 0.58 | 3.19 (41) | <0.01 | 4.21 (41) | <0.01 | -2.55 (41) | 0.02 | -2.62 (41) | 0.01 |

Numbers in parentheses represent degrees of freedom.


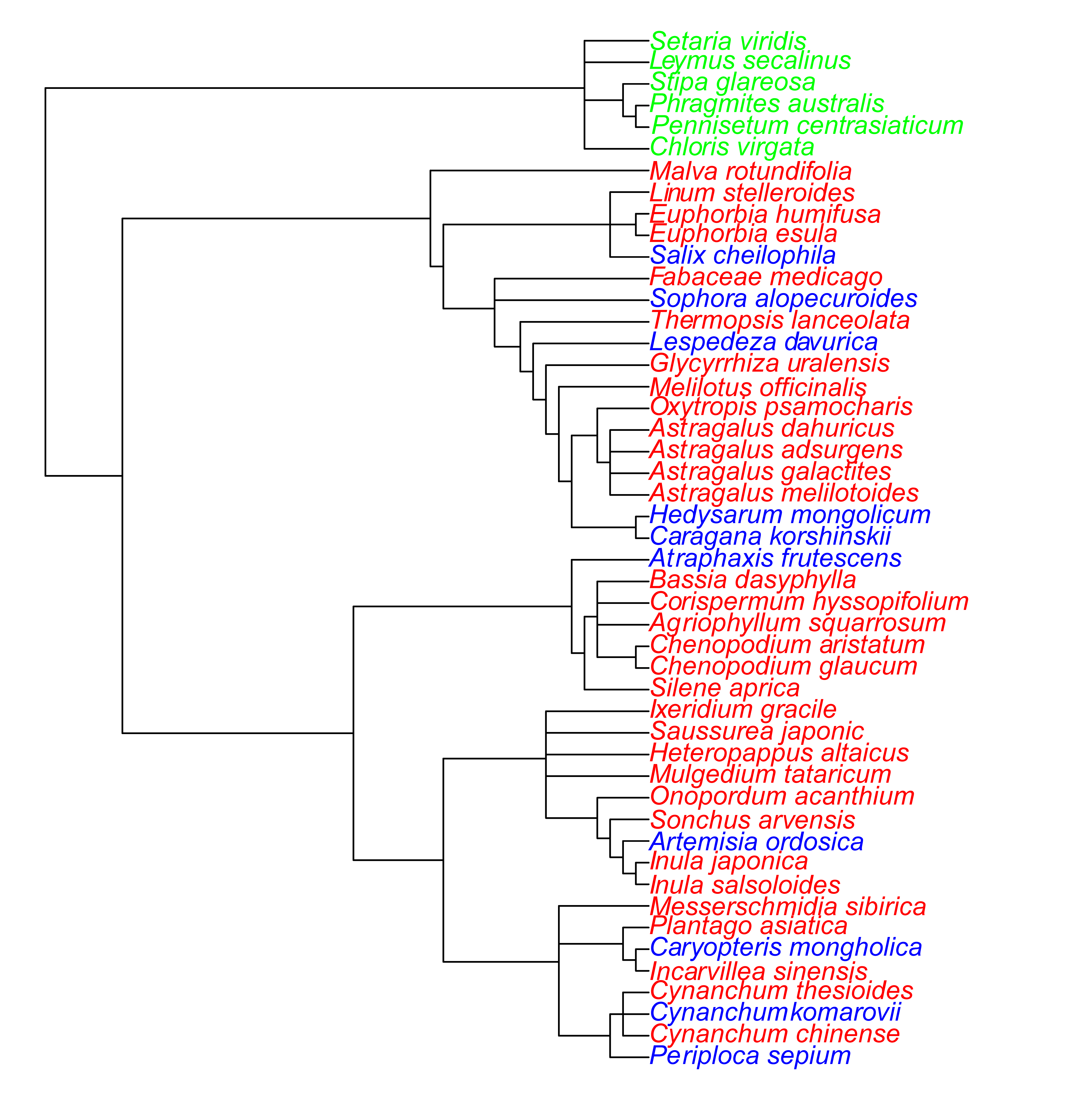


**Supplementary Figure 1.** Phylogeny of the 48 investigated species. Green for grasses, red for forbs, and blue for woody species.


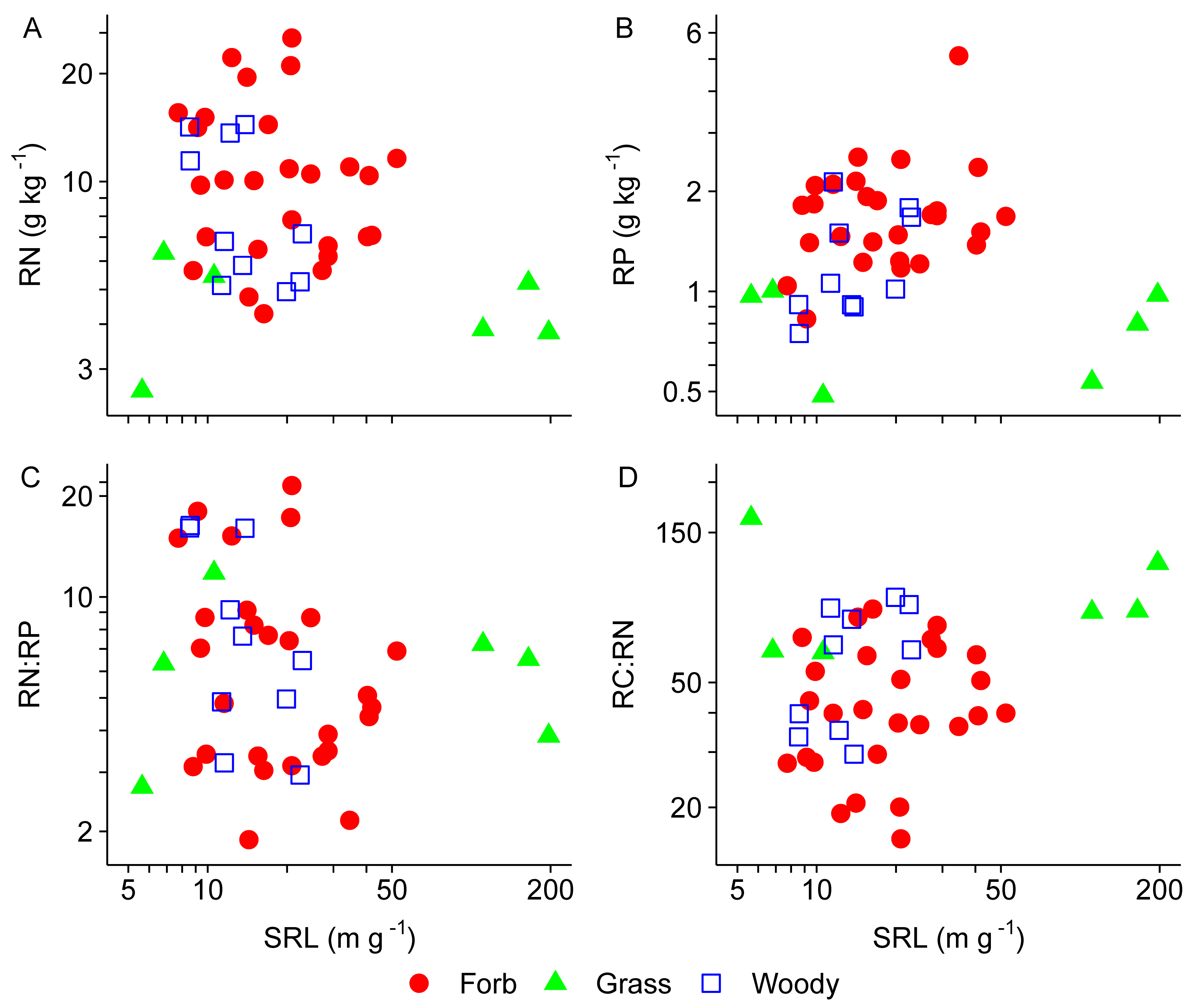


**Supplementary Figure 2.** Relationships between specific root length (SRL) and other fine root traits across species. RN, fine root nitrogen content; RP, fine root phosphorus content; RN:RP, fine root N:P ratio; RC:RN, fine root C:N ratio. Species means are shown for forbs (closed circle), grasses (triangle), and woody species (open square). The log_10_ scale was used on both x- and y-axis.


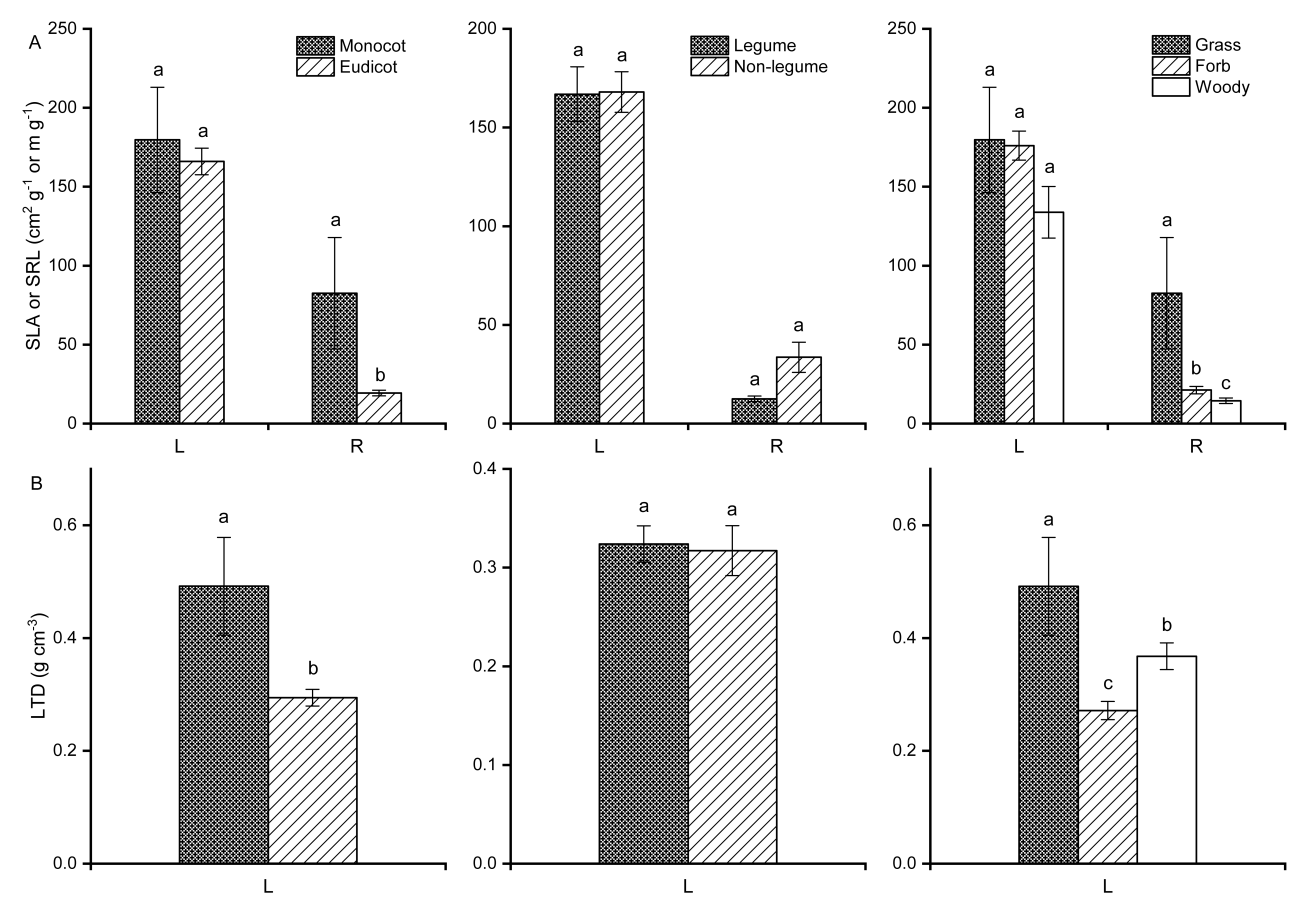


**Supplementary Figure 3.** Comparisons of (**A**) SLA or SRL and (**B**) tissue density of leaves (LTD) among functional groups. Data are shown as mean ± standard error of mean. L and R represent leaf and root, respectively. Lower-case letters denote pairwise contrasts at the 0.05 level (the Tukey HSD method).


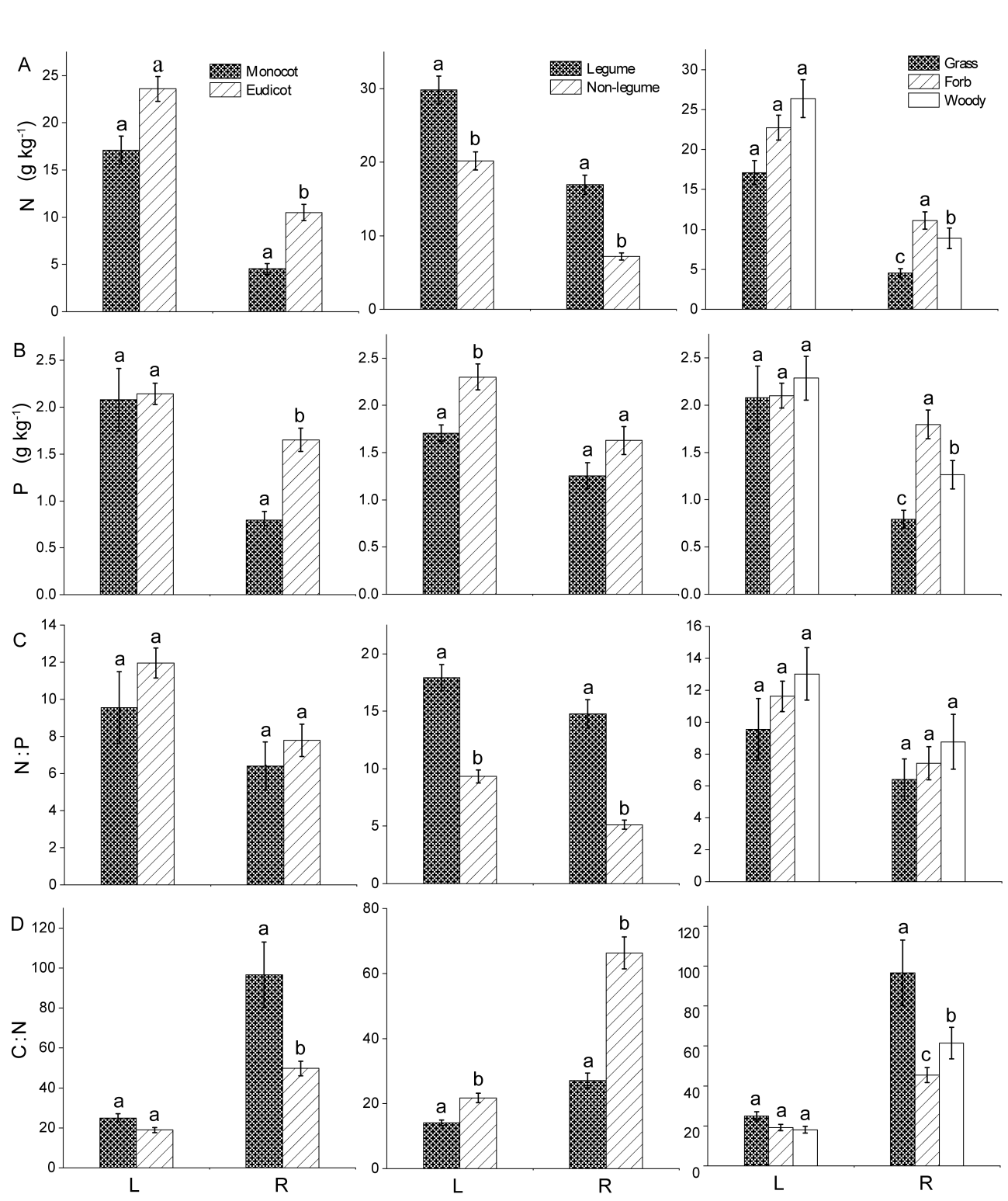


**Supplementary Figure 4.** Comparisons of (**A**) N content, (**B**) P content, (**C**) N:P ratio, and (**D**) C:N ratio among functional groups. Data are shown as mean ± standard error of mean. L and R represent leaf and fine root, respectively. Lower-case letters denote pairwise contrasts at the 0.05 level (the Tukey HSD method).
